# Lower prostate cancer risk in Swedish men with the androgen receptor E213 A-allele

**DOI:** 10.1007/s10552-017-0859-1

**Published:** 2017-02-07

**Authors:** Magdalena Bentmar Holgersson, Yasir Ruhayel, Magnus Karlsson, Aleksander Giwercman, Anders Bjartell, Claes Ohlsson, Dan Mellström, Östen Ljunggren, Mohammad-Ali Haghsheno, Jan-Erik Damber, Yvonne Lundberg Giwercman

**Affiliations:** 1 Department of Translational Medicine, Lund University, Jan Waldenströms gata 35, Building 91, Plan 10, 205 02 Malmö, Sweden; 2 Department of Urology, Skåne University Hospital, Malmö, Sweden; 3 Department of Orthopedics and Clinical Sciences, Lund University, Malmö, Sweden; 4 Departments of Internal Medicine and Clinical Nutrition and Geriatrics at the Sahlgrenska Academy, Center for Bone and Arthritis Research, Gothenburg University, Gothenburg, Sweden; 5 Department of Medical Sciences, University of Uppsala, Uppsala, Sweden; 6 Department of Urology, Sahlgrenska Cancer Center, Institute of Clinical Sciences, Sahlgrenska Academy at the University of Gothenburg, Gothenburg, Sweden

**Keywords:** Androgen receptor, Prostate cancer, Genetic variants

## Abstract

**Background:**

In a previous population-based study on 3369 European men with self-reported prostate cancer (PCa), it was shown that androgen receptor (*AR*) haplotype designated *H2* was associated with high levels of serum PSA (prostate-specific antigen) concentration, and, at the same time, with low risk for PCa. The aim of this study was to replicate this finding in other cohorts, with registry-based cancer diagnosis.

**Methods:**

Using data from two population-based cohorts; the Malmö Diet and Cancer Study (MDCS, *n* = 12,121) and the Swedish Osteoporotic fractures in men study (MrOS, *n* = 1,120), 628 men with PCa and 1,374 controls were identified and genotyped. PCa data were collected from the Swedish national cancer registry. PCa odds ratios (ORs) and 95% confidence intervals (CIs) were calculated for carriers of the particular *AR* haplotype, tagged by the *rs6624304 T*-allele.

**Results:**

The 15% of men who were carriers of the *AR* haplotype *H2* had approximately one-third lower risk for PCa diagnosis compared to those with the most common *H1* variant (OR 0.65; 95% CI 0.45–0.94; *p* = 0.021). The same trend, although not statistically significant (OR 0.75; 95% CI 0.47–1.24; *p* = 0.275), was observed in MrOS Sweden. When both cohorts were merged, an even more significant result was observed (OR 0.68; 95% CI 0.51–0.90; *p* = 0.008).

**Conclusions:**

Swedish men with the variant *AR* haplotype *H2*, tagged by *rs6624304*, have significantly lower risk of PCa compared to those with the more common variant.

## Introduction

Among European men, prostate cancer (PCa) is the most common non-skin cancer, and it is the third most common cause of cancer-related mortality [1]. It is well established that PCa is hormone-dependent, and androgen deprivation therapy with pharmacologic or surgical castration is, therefore, a corner stone in the treatment of advanced PCa.

Androgens mediate their effects by altering gene expression via the androgen receptor (AR), a hormone-induced transcription factor. One gene that is dependent on ligand activated AR for its expression is *Kallikrein-related peptidase 3*, encoding for the serine protease known as prostate-specific antigen (PSA).

The *AR* gene harbors two tri-nucleotide repeats in the coding region; the extensively studied *CAG*-repeat, which encodes for a varying number of the amino-acid glutamine, and the *GGN* repeat, encoding for a chain of glycines [2–4]. Apart from the repeats, the *AR* also holds a single-nucleotide polymorphism (SNP) in its first exon (*rs6152*) located in between the *CAG*- and the *GGN*-repeats. This variant is a synonymous *GAG* > *GAA* substitution in codon E213 and is also sometimes referred to as StuI as the presence of the *G*-allele allows for digestion with the restriction enzyme *Stu* I [5].

We have recently, in the community-based European Male Ageing Study (EMAS), consisting of 3369 men, between 40 and 80 years of age and from eight European centers, identified two different *AR* haplotypes [6]. The 16% of men with the minor haplotype (designated *H2*), to which the minor *A*-allele of E213 belongs, exhibited a nearly twofold increased risk of having serum PSA above 3 and 4 ng/mL, which are clinically used cut-off values for further investigation on the suspicion of PCa. Yet, these men were only one-third as likely to be diagnosed with PCa after 4 years of follow-up compared to subjects carrying the *G* allele (*H1*). However, as the number of PCa cases was relatively small and PCa self-reported, the objective of current work was to replicate the previous study regarding PCa risk in two other cohorts, with cancer cases collected from the Swedish National Cancer Registry.

## Materials and methods

### Study design

The study was based on two cohorts:

1. The Malmö Diet and Cancer Study (MDCS), from which a nested case–control population consisting of PCa cases and controls was extracted [7];
2. The Malmö and Gothenburg parts of the Swedish Osteoporotic fractures in men study (MrOS), which is a prospective study on osteoporosis in men [8].

Men with sufficient amount of DNA in the bio-bank had been genotyped regarding SNPs in the AR gene. These SNPs were designed to capture all variation in the gene.

Written informed consent was obtained from all participants. The study was approved by the ethical committee board at Lund and Gothenburg Universities, respectively.

### MDCS, nested case–control study

**Fig. 1:**
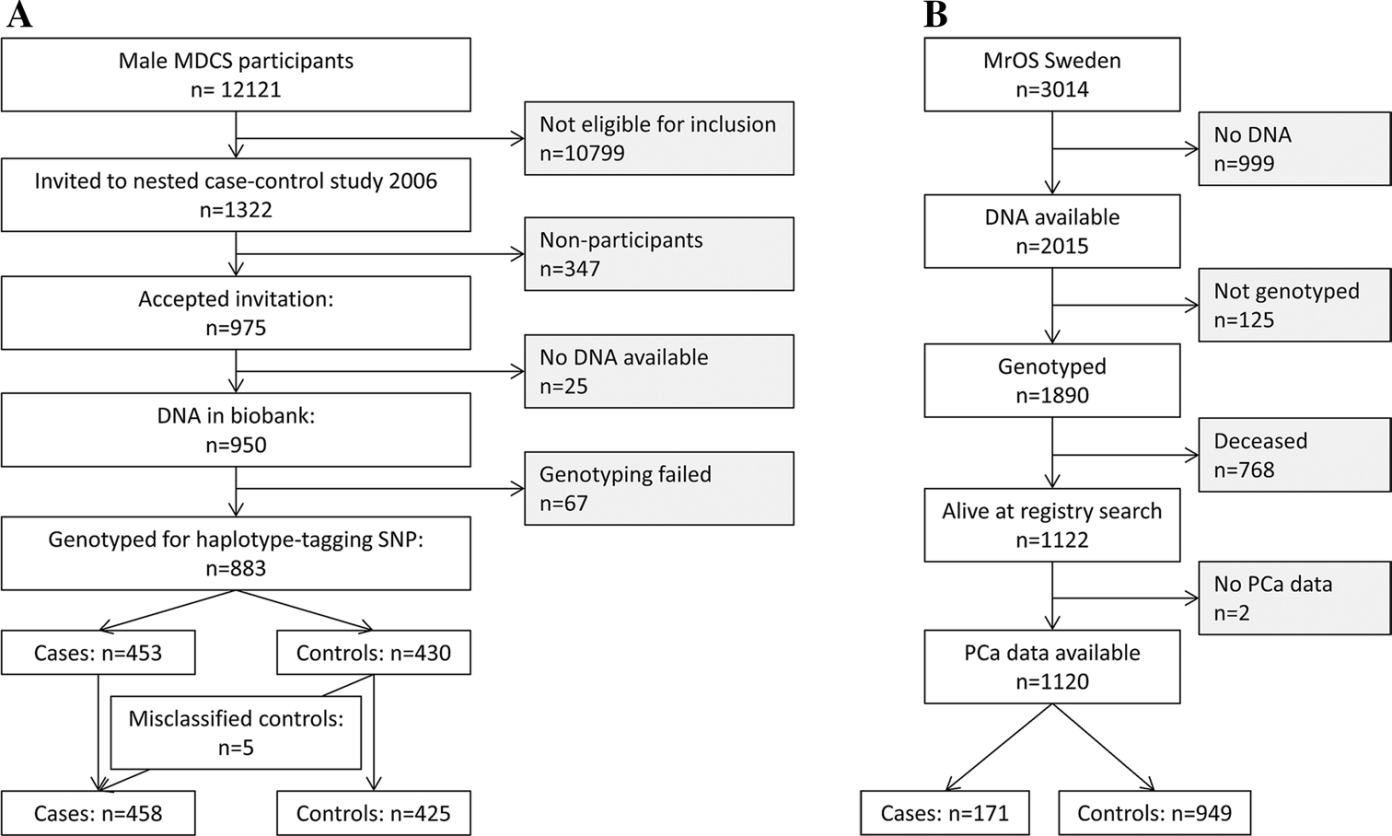
Flow charts describing the exclusion process in the cohorts **a** MDCS and **b** MrOS

In the period 1991–1995, men born between 1923 and 1945 (*n* = 31,514) and women (*n* = 42,624) born 1923–1950 and living in the city of Malmö, Sweden were invited to participate in a prospective study on western diet and lifestyle factors in relation to cancer [9]. The only exclusion criteria were mental incapacity or insufficient Swedish language, leaving 68,905 eligible subjects of which 28,873 were men. Of these, 12,121 men accepted the invitation to participate in the MDCS study, 38% of the target male population (Fig. 1a).

Using the Swedish National Cancer Registry, 661 prevalent cases of PCa in the MDCS cohort who were still alive as of 2006 were identified; 5% of the enrolled men. Each man with PCa was matched with a control based on age (±90 days) and date of enrollment (±90 days) resulting in 1,322 eligible men. Of these, 975 men (74%) agreed to participate in a nested case–control study, initiated for a study regarding PCa in relation to number of children [7]. Those who accepted the invitation and those who did not respond to the invitation were of similar age, with the mean age and standard deviation (SD) being 74.3 years (SD ± 5.7) and 75.4 years (SD ± 6.0), respectively. Of the participants, 25 were excluded due to insufficient DNA quantity in the bio-bank, while genotyping failed in 13 cases. Five controls were discovered as misclassified and, therefore, transferred to the group of PCa cases, giving a total number of 458 cases and 425 controls that were genotyped. Of these, 421 (89%) and 389 (89%), respectively, reported that they were born in Sweden. The 68 men born outside of Sweden represented 20 countries, with most men born in Denmark (*n* = 10), Germany (*n* = 7), Hungary (*n* = 5), Yugoslavia (*n* = 4), and Norway (*n* = 3). The number of men who had not reported birth-country was 32.

### The Swedish MrOS study

The Swedish part of the MrOS Study, originally initiated to study osteoporotic fractures in men, consisted of 3014 men, aged 69 to 81 years (mean age at inclusion 75.4 years) from three centers (Malmö, *n* = 1005; Uppsala, *n* = 999; and Gothenburg, *n* = 1010). These men were randomly identified using national population registries and enrolled between October 2001 and December 2004. To be eligible for the study, the participants had to be able to walk without aid and able to complete a questionnaire. There were no other exclusion criteria [8]. The inclusion rate at baseline for the Swedish part of MrOS was 45%. Only the subpopulations from Malmö and Gothenburg had been genotyped and, therefore, *n* = 2015 men from the Swedish MrOS study were eligible for inclusion (Fig. 1b).

Since the prerequisite for both the previous study on European men using the EMAS cohort and the MDCS was that the men were alive at endpoint, only the men in the MrOS that were alive when the cancer data were accessed from the National Prostate Cancer Registry in December 2013 (mean time to registry search 10.4 years) were included. Genotype data were available for 1120 of these men (59%); mean age at registry search was 84.2 years (SD ± 2.9). Both prevalent and incident prostate cancers were included in the analyses (*n* = 171).

The genotyping of the MrOS subjects was based on blood sampling performed at the initiation of the study. By assessing the distribution of *H1* and *H2* haplotypes among the768 men who had died between the start of the MrOS study and the registry search in December 2013, selection bias was estimated.

### Genotyping

The HapMap database (Release 27) was used to select haplotype-tagging SNPs (htSNPs) capturing all variation (with *R* ^2^ > 0.80) within the gene and 20 kilobases upstream and downstream of it, using the Tagger Multimarker algorithm and restricting htSNPs to those with a minor allele frequency (MAF) >5% in the HapMap CEU samples (http://www.hapmap.org).

The nested MDCS samples genotyping was performed using the Sequenom iPLEX Gold assay and MassARRAY matrix-assisted laser desorption/ionization time-of-flight (MALDI-TOF) platform in accordance with the manufacturer’s instruction (Sequenom Inc., San Diego, CA, USA), whereas the MrOS samples were analyzed by matrix-assisted laser desorption ionization–time-of-flight mass spectrometry on the Sequenom MassARRAY platform (San Diego, CA, USA). Assays were performed with randomly sorted samples and controls.

Genotype information was collected from the European population (EUR) in the phase 1 data from the 1000 genomes project (http://www.ensembl.org/). Genotype data on the SNPs used in EMAS and in MrOS/MDCS were pairwise calculated regarding linkage disequilibrium and for identification of SNPs with the highest haplotype predictability.

When the SNPs used in current study were compared with the SNPs used in the previous EMAS cohort, *rs6624304* had the highest haplotype predictability (*r* ^2^ range; 0.889–0.944), where the *T*-allele belonged to the PCa low-risk haplotype *H2*. In other words, in the European subpopulation of 1000 genomes, the allele of the haplotype marker used in the EMAS (*rs1204038*) could be predicted by the allele in *rs6624304* in 99% of the subjects. All the SNPs that in the EMAS cohort were found to be in high linkage disequilibrium, quantified as high *r* ^2^ values, and were also in the EUR subpopulation of 1000 genomes found to have high *r* ^2^.

### Statistics

Logistic regression was used to calculate the odds ratio (OR) and 95% confidence interval (CI) for having PCa in relation to the *rs6624304* genotype. All calculations were performed both with and without age adjustments. In the MDCS study, calculations were done for the whole cohort as well as for those born in Sweden only.

Only men alive in December 2013 were used in the calculations on PCa risk in the MrOS cohort. For this cohort, in addition, the genotype-related OR for dying during the period between study initiation and December 2013 was calculated. Since cause of death was not available, to rule out that carriers of one haplotype had a more aggressive PCa than the other one, the number of days between PCa diagnosis and death was investigated using linear regression. In addition, for the included cases, tumor data (Gleason, PSA, Metastasis, Nodes, Tumor stage) at the time of diagnosis for the two haplotypes were compared using Mann Whitney *U* test and Pearson’s Chi-square test.

Comparison of haplotype frequencies in the different cohorts to the European subpopulation from 1000 genomes was done using Fisher’s exact test. All calculations were performed in SPSS 22 Software (SPSS, Inc., Chicago, USA).

## Results

### Haplotype distribution compared to European 1,000 genomes cohort

In the current study, as in the four subpopulations of 1,000 genomes, the *H1* haplotype was the most common haplotype in European populations. *H1* is also the only haplotype present in East Asian populations, while *H2* is the most common in the African populations (Table 1).

**Table 1:** Frequency of each haplotype, represented by the allele frequencies in *rs6624304*, in the reported PCa cases and controls, as well as the 1000 genomes subpopulations, *n* (%)

	MDCS	MrOS	AMR	AFR	ASN	EUR
*H1; rs6624304C*	747 (85)	951 (85)	218 (81)	32 (10)	428 (100)	495 (86)
*H2; rs6624304T*	136 (15)	169 (15)	51 (19)	283 (90)	0 (0)	79 (14)

The allele frequencies in MDCS and MrOS did not differ from the European 1000 genomes subcohort (*p* = 0.406 and *p* = 0.514, respectively).

### Prostate cancer risk

The frequency of prevalent PCa was higher at inclusion in MrOS than in MDCS (9 vs 5%).

In the MDCS, the minor haplotype *H2* was associated with 35% decreased odds for PCa, in all men as well as in those born in Sweden only (Table 2).

**Table 2:** Frequency of *rs6624304* in PCa cases and controls

Study Cohort	PCa, *n* (%)	Controls, *n* (%)	OR (95% CI)	*p*	OR (95% CI)^a^	*P* ^a^
MDCS						
*rs6624304* ^b^						
C	400 (87)	347 (82)	Reference	0.020*	Reference	0.021*
T	58 (13)	78 (18)	0.65 (0.45–0.93)		0.65 (0.45–0.94)	
MDCS^c^						
*rs6624304* ^b^						
C	356 (87)	309 (82)	Reference	0.022*	Reference	0.023*
T	51 (13)	70 (18)	0.63 (0.43–0.94)		0.64 (0.43–0.94)	
MrOS						
*rs6624304* ^b^						
C	150 (88)	801 (84)	Reference	*0.266*	Reference	*0.275*
T	21 (12)	148 (16)	0.76 (0.47–1.24)		0.75 (0.47–1.24)	
Merged						
*rs6624304* ^b^						
C	550 (87)	1148 (84)	Reference	0.025*	Reference	0.008*
T	79 (13)	226 (16)	0.73 (0.55–0.96)		0.68 (0.51–0.90)	

Odds ratios for PCa calculated with the major allele as reference

*Significant at the 0.05 level

^a^Adjusted for age at inclusion in the current study (MDCS) or age at examination (MrOS)

^b^Marker for *H1* and *H2*, and *C*- and *T*-allele, respectively

^c^Swedish

In the MrOS subjects, the same tendency, although without reaching the level of statistical significance, was observed.

In the merged cohort, the odds for being diagnosed with PCa were 32% lower in carriers of *H2* as compared to those with *H1*.

Tumor characteristics at diagnosis did not differ between the carriers of the different haplotypes (Table 3).

**Table 3:** Comparison of tumor characteristics for the two alleles of rs6624304

	*rs6624304C, n* (%)	*rs6624304T, n* (%)
PSA (ng/µl)		
Unknown, *n* (% of cases)	244 (44)	24 (30)
PSA median (25–75 percentile)	9.15 (5.60–16.00)	8.40 (5.90–17.10)
PSA < 10, *n* (%)	162 (53)	34 (62)
PSA 10-19.9, *n* (%)	82 (27)	12 (22)
PSA > 20, *n* (%)	62 (20)	9 (16)
Gleason score		
Unknown, *n* (% of cases)	122 (22)	17 (22)
Gleason median (25–75 percentile)	6 (6–7)	6 (6–7)
Gleason < 7	247 (58)	34 (55)
Gleason 7	134 (31)	18 (29)
Gleason > 7	47 (11)	10 (16)
Tumor stage		
Unknown, *n* (% of cases)	63 (11)	8 (10)
T0	4 (1)	0 (0)
T1	225 (46)	29 (41)
T2	170 (35)	28 (39)
T3	82 (17)	14 (20)
T4	6 (1)	0 (0)
Nodes		
Unknown, *n* (% of cases)	417 (76)	59 (75)
N0	128 (96)	19 (95)
N1	5 (4)	1 (5)
Metastasis		
Unknown, *n* (% of cases)	266 (48)	37 (47)
M0	275 (97)	41 (98)
M1	9 (3)	1 (2)

### Mortality risk in relation to haplotype

In the 768 deceased men, 660 (86%) and 108 (14%) were carriers of *H1* and *H2*, respectively. There were no significant differences in mortality between the groups, neither before nor after adjustment for age at inclusion, OR (95% CI) for *H2*: 0.90 (0.69–1.18), *p* = 0.444 and 0.92 (0.71–1.20), *p* = 0.546, respectively. Of the deceased men with a PCa diagnosis, *n* = 168 had been genotyped for the *AR* variant. For these men, the number of days between diagnosis and all-cause death did not differ between the two haplotypes (mean (SD) H1: 2,871.01 (2,119.52), H2: 3,008.32 (1,343.00), *p* = 0.742; adjusted for age at inclusion: *p* = 0.765).

## Discussion

The previous finding regarding an association between *AR* haplotype (*H2*) and low-risk for Europeans for being diagnosed with PCa was replicated in the current study. Although the same tendency was observed in both cohorts investigated, it was statistically significant in the larger MDCS, but not in the MrOS, most probably due to the circumstance that the MrOS subcohort was smaller and the statistical power, therefore, relatively low. Another explanation could be selection bias, with a higher proportion of men with PCa assigning for participation in MrOS. It is well known that a cornerstone in treatment of PCa is androgen ablation, and androgen deficiency, in turn, is a risk factor for osteoporosis. Of the men with PCa, up to 53% are affected by this bone disorder [10]. These men could, therefore, be more inclined to participate in a prospective study on osteoporosis when contacted than men without PCa. However, this circumstance would not affect the frequencies of the genetic variants.

Our findings have interesting clinical and biological implications. We have previously reported that the *H2* carriers without PCa have a 65% higher odds of having PSA levels above the level of 3 or 4 ng/mL, which generally are used as cut points for referral to urological examination, including prostate biopsies [6]. Although no PSA data were available for the participants without PCa in the cohorts included in this study as they were from the general population and not originally participating in a study on PCa, the combination of a lower risk of manifest PCa in a group of men previously found to be more likely to present with high PSA due to a certain inherited gene variant calls for efforts to develop genotype adjusted normal ranges for this cancer marker. It is possible that inclusion of the SNP *E213* could improve the performance of the PSA-test.

When tumor characteristics were compared for the two haplotypes, no differences in aggression or stage were found. However, many cases were missing detailed tumor data. Tumor stage, the most complete variable (10% missing values), did not indicate any overrepresentation of men in any category.

In a previous study on PCa, on men from the Cancer Prostate in Sweden (CAPS) cohort, those with the *A*-allele of *rs6152*, part of our minor *H2*-haplotype, had a significantly reduced risk of developing PCa [11]. However, when the study was replicated in seven pooled cohorts (5 American and 2 European), the variant was no longer associated with reduced PCa risk [12].

The haplotype reported by us as implying lower PCa risk appears to be the most common haplotype in African populations, while the high-risk haplotype is the only haplotype in East Asia. This is contradictory to the incidence of PCa in these populations, as several studies have reported higher risk of PCa in African-American men in comparison with Caucasians and the incidence of PCa is much lower in Asian countries than elsewhere [13]. This indicates that the biological mechanisms linking this particular *AR* haplotype to the risk of PCa are highly dependent on other factors, of which some can be lifestyle-related, whereas others are likely to be genetic. This could also explain why the study by Lindström et al. [12] failed to repeat the results in the larger data set consisting of a more genetically diverse population, where population stratification bias could dilute the effect of the genetic variants.

This contradictory relationship between haplotype distribution and population risk also seems to apply for androgenic alopecia. Studies regarding this condition have reported alleles included in the *H1* haplotype to be associated with increased risk of male pattern baldness [14–18]. In addition, for this condition, although the highest frequencies of risk alleles are found in Asian men, their risk of male baldness is 50% lower than among Europeans [15]. A meta-analysis comprising more than 2000 men from several countries found that the association between the E213 variant that belongs to the *H2* haplotype and baldness was applicable for Caucasians only [18].

A limitation of our study was that only men who were alive at follow-up were included. Since age at endpoint was relatively high in both cohorts, information may have been lost due to exclusion of men with a shorter life-span or more aggressive early onset PCa. However, in the current study, there was no significant difference in all-cause mortality between the men with the two haplotypes during the 10-year follow-up period and no difference in the number of days between diagnosis and all-cause death in the deceased men with PCa. Furthermore, finding of similar distribution of the two haplotypes in the current study as in Europeans in the 1000 genomes study speaks against significant selection bias.

Another point of criticism could be the use of different SNPs as markers of the *H1*/*H2* haplotype in EMAS (*rs1204038*) and in the current study (*rs6624304*). However, we do not consider this as a significant problem as the former SNP can be predicted by the allele in the latter in 99% of the cases in European cohorts.

The strength of the study was access to the Swedish National Cancer Registry, which is a mandatory registry of all cancers in Sweden with an approximated completeness of registration of 99%, ensuring a complete assessment of PCa diagnosis. Moreover, the current study included approximately ten times as many PCa cases compared to our previous study on European men (EMAS), and, also, in the current study, men with the *H2* haplotype had one-third lower odds of being diagnosed with PCa compared to counterparts with the most common haplotype *H1*.

In summary, based on two large Swedish community-based populations, we have confirmed the previous indication that 15% of European men who are carriers of the second most frequent *AR* haplotype have approximately one-third lower odds of PCa than counterparts with the most frequent *AR* variant.
